# General practitioners' use and experiences of palliative care services: a survey in south east England

**DOI:** 10.1186/1472-684X-7-18

**Published:** 2008-11-05

**Authors:** Sabrina Bajwah, Irene J Higginson

**Affiliations:** 1 Trinity Hospice, 30 Clapham Common North Side, Clapham, SW4 ORN, London, UK; 2 Department of Palliative Care and Policy, King's College, London, UK

## Abstract

**Background:**

The role of the General Practitioner (GP) is central to community palliative care. Good liaison between the different professionals involved in a patient's care is extremely important in palliative care patients. In cases where GPs have previously been dissatisfied with palliative services, this may be seen as a barrier to referral when caring for other patients. The aim of this survey is to investigate the use and previous experiences of GPs of two palliative care services, with particular emphasis on barriers to referral and to explore issues surrounding the GP's role in caring for palliative patients.

**Methods:**

Design: Descriptive postal survey of use and experience of palliative care services with particular emphasis on barriers to referral. Setting: One Primary Care Trust (PCT), south London, England, population 298,500. Subjects: 180 GPs in the PCT, which is served by two hospice services (A&B).

**Results:**

An overall questionnaire response rate of 77% (138) was obtained, with 69% (124) used in analysis. Over 90% of GPs were satisfied with the palliative care services over the preceding two years. Two areas of possible improvement emerged; communication and prescribing practices. GPs identified some patients that they had not referred, most commonly when patients or carers were reluctant to accept help, or when other support was deemed sufficient. Over half of the GPs felt there were areas where improvement could be made; with clarification of the rules and responsibilities of the multi disciplinary team being the most common. The majority of GPs were working, and want to work with, the specialist services as part of an extended team. However, a greater number of GPs want to hand over care to the specialist services than are currently doing so.

**Conclusion:**

A large number of GPs were happy with the service provision of the palliative care services in this area. They suggested that 3 out of 4 terminally ill patients needed specialist input. Views of services were largely positive, and reasons for non referral were unrelated to previous experience of the specialist services.

## Background

The role of the general practitioner (GP) is central to community palliative care [1]. Structurally, the position of GPs as the first point of entry to and liaison with other, more specialist services has become more important with the increased emphasis upon community care [2]. The experience of GPs of palliative care services vary in the literature, Boyd et al [3], in the East End of London found a small number of GPs 14/187 (7%), commented on inadequate communication and poor cooperation between themselves and the home care team. However, a questionnaire study of GPs in rural North Wales, found that 71% of GPs thought that communication between professionals caring for terminally ill patients was described as very good or good [4].

Seamark et al [5] found that 10% of the 71 GPs replying to their questionnaire survey in Exeter felt that when the specialist service was involved, they found it difficult to know who had overall responsibility for the patients care and their contribution felt underrated (6%). However, only 71 of the 121 GPs responding to the questionnaire had knowledge of the hospice service. Shipman et al [6] also discussed the perception of a split between responsibility for prescribing and lack of control over decision making. They felt this may have led to negative perceptions by GPs about collaborative working. It is not clear how many of the GPs this included.

The exclusion of GPs by specialist nurses was noted in the study by Field et al [2], as was the dissatisfaction of the GPs to the lack of recognition as the primary carer by Desmedt et al in Belgium [7].

The use of specialist palliative care services varies. Shipman et al [6] found 9 out of 63 GPs interviewed seldom used specialist services and only 8% of the GPs surveyed worked with the specialist services as part of an extended team. Field et al [2] reported GPs wanted to be able to call in expert help as required but not to surrender care to the palliative care services. However, in the survey by Boyd et al [3], 18% of the GPs responding wanted the specialist services to take over care of the patient, with shared medical care favoured by 75% of general practitioners.

In cases where GPs had previously experienced difficulty in accessing help, this may be seen as an absolute barrier to contact with the service when caring for other patients [6]. In the US, one quarter (24%) of primary care physicians expressed concerns about loss of contact with patients and timely communication with hospice providers [8]. This was then seen as a barrier to future referral. Similarly, Shipman et al [6] found that nine out of sixty three GPs interviewed seldom used specialist services; the one key reason sited was previous poor communication. Communication problems with other health professionals were also perceived as a barrier to delivering palliative care on a day to day basis by GPs in the Netherlands [9].

This paper surveyed, as part of SB's MSc in palliative care, the views of GPs on how they are using and how they would like to use palliative care services. The survey also aimed to determine the experience of palliative care services and if these affected referral behaviour.

## Methods

The Primary Care Trust (PCT) was in outer London, south England, with a population of 298,500; 48% were male, 19.3% of retirement age, similar to the national average for England and Wales (18.5%). According to the 2004 English Indices of Multiple Deprivation, the PCT ranked 238 out of 354 local authorities (the most deprived = 1), with a Standardised Mortality Ratio better than average of 89 in 2003 (Office of National Statistics). The number of cancer deaths in the preceding two years (Dec 2001–2003) was 1,292, of which 870 were at home. (PCT, personal communication April 2004). One hundred and eighty general practitioners were responsible for the palliative care needs of the patients in the PCT (PCT personal communication April 2004). There were 2 hospices (called A and B for this report) offering palliative care where required dependent on the postcode of the patient. Hospice A covers the PCT only, but Hospice B's catchment area covers 5 London Boroughs, looking after 1,800 patients and families each year. Hospice B is significantly larger than Hospice A. Both hospices serve the PCT through their home care services, offering 24 hour support and advice in caring for terminal patients in the community. There is an in-patient facility available at Hospice B to which all patients in the PCT can be referred. Alternatively, patients may also receive palliative care in the acute hospital setting.

Patients were initially referred to the service by the GP, hospital doctor or district nurse with subsequent collaboration with the specialist service in caring for these patients. Both services aimed to work closely with the GPs in caring for these patients.

A list of the GP principals in the PCT was obtained from the local authority. Each practice was called individually to confirm the doctors working there, and to ensure that all salaried doctors were included. Locums were excluded due to the temporary nature of their practice.

A postal questionnaire was sent out to the total population of permanent GPs whose patients were potential users of either Hospice A or Hospice B's home palliative care teams and was developed from previous questionnaires and research used by Cartwright [10], Shipman et al [6], Desmedt et al [7], Ogle et al [8] and Todd et al [11].

The questionnaire covered demographics of GPs, numbers referred to the two palliative care services, how GPs are using and how they would like to use the service, satisfaction levels of the service in the past and areas for further improvement. Questions were fixed choice – yes, no and not applicable response categories. The questionnaire was limited to two sides of A4 to improve response rates and minimise non-response bias. The final questionnaire was sent out to the 180 GPs in the PCT. A covering letter explaining that SB was a GP registrar working in the area and a stamped addressed return envelope was enclosed with the questionnaire. A reminder was sent after one month to non-responders with a follow up reminder for GPs still not responding after two months.

Data collected was tabulated by variable, using Statistical Package for the Social Sciences (SPSS for Windows, Version 10.0). Results of demographics obtained were compared to FHSA figures for October 2004 of the GP partners in the area (PCT, personal communication November 2004). Frequency analysis was used to generate descriptive statistics for categorical variables. Chi-squared tests were used, significance levels taken at p =< 0.05.

Research approval was sought and granted by the LREC and RND committees (LREC reference 746). All responses were treated with confidentiality and replies were coded to allow identification of non-responders only.

## Results

### Response rates

138 out of 180 GPs completed or replied to the questionnaire, overall response rate of 77% and 124 (69%) completed the questionnaire (Table 1). 42 GPs did not respond despite 3 questionnaires being sent over a 3 month period. The number of questionnaires available for analysis was 124 (69%).

**Table 1:** Response rates for questionnaire

Questionnaire completed	124
Away on sick leave	1
No experience and unable to comment	6
Did not want to take part in questionnaire	7
No response	42

### Respondent demographics

Compared to the health authority figures the responding GPs were similar in age and gender. However, single handed practices were under-represented, as were small practices of less than 5,000 patients. Practices with list sizes of 5,000 – 10,000 were over-represented, as were trainer GPs. All 17 trainers in the area responded to the questionnaire – comprising 14% of the overall response (Table 2).

**Table 2:** Demographics of GPs responding compared with those in the area

**Demographics**	**N(%) of GPs responding**
**Gender**	
Male	67 (54%)
**Age**	
<35 years	17 (14%)
35–44 years	38 (31%)
45–54 years	47 (38%)
55+	19 (15%)
Missing data	3(2%)
**No. of partners**	
1	5(4%)
1.5–3	46(37%)
>3–<6	39(32%)
6+	30(24%)
Missing data	4(3%)
**Years as GP**	
1–5	28(23%)
6–10	16(13%)
11–15	17(14%)
16–20	24(19%)
>20	33(27%)
Missing data	6(5%)
**Practice size**	
<5000	15(12%)
5–10,000	77(62%)

### How GPs are managing palliative care patients

Most GPs (n = 80, 64%) felt that fewer than 1 in 4 palliative care patients could be managed without the help of the specialist services. There was no relationship between the proportion of palliative care patients that the GP felt could be satisfactorily managed without the help of the specialist services and sex, age, number of partners in practice, years as GP, practice size and number of terminal patients cared for in the last two years. There was however, a significant relationship with trainer status, although the numbers were small; 53% (n = 9) of trainers felt that 25% or more of patients' symptoms could be controlled without the help of the specialist services compared to 28% (n = 26) of non trainers (χ^2 ^= 4.26, df = 1, p = 0.04).

### How GPs are using and would like to use the two services

78 of the GPs had used Hospice A and 89 had used Hospice B for their patients (Table 3), mainly by working with them as part of an extended team. GPs wanted to work more as part of an extended team but to handover more care than they were doing.

**Table 3:** How GPs are using and would like to use the two palliative care services

	Hospice A		Hospice B	
	n(%)	missing data (n)	n(%)	Missing data (n)
Seldom using service	6 (8)	4	7(8)	3
Would like to seldom use service	1 (1)	7	2(2)	5
Using service as a resource	31(40)	4	42(47)	3
Would like to use service as a resource	22(30)	7	35(40)	5
Working with service as part of an extended team	55(71)	4	59(66)	3
Would like to work with service as part of an extended team	57(76)	7	58(67)	5
Handing over care	20(26)	4	39(44)	3
Would like to hand over care	28(37)	7	38(44)	5

### What GPs would like to see more of

The most common request for improvement was for clarification of the rules and responsibilities of the Multidisciplinary Team (MDT) (Table 4) A smaller number of GPs wanted the specialist services to identify the level of support required by the GP, increased participation in decisions whether to hospitalise and increased participation in decisions concerning treatment. A number of GPs (ranging 38–50% of users) did not feel that any of these factors needed to be improved.

**Table 4:** What GPs would like to see more of from the two hospice services

	Hospice A		Hospice B	
	n (%)	Missing data N	n (%)	Missing data N
Recognition of GP as primary carer	16(22)	10	10(12)	7
Participation in decisions concerning treatment	21(30)	10	18(22)	7
Participation in decisions whether to hospitalise	13(18)	10	20(24)	7
Clarification of rules and responsibilities of the MDT	28(40)	10	24(28)	7
Identification of level of support required by GP	18(26)	10	22(26)	7
None of the above	27(38)	10	44(50)	7

### Experience of palliative care services

Both hospices scored highly in the satisfaction levels (ranging 90–94%) as shown in Table 5. This included experience of practical support, guidance and level of involvement. There was some dissatisfaction regarding communication and prescribing practices.

**Table 5:** Satisfaction ratings of GPs using Hospice A and B

		Very satisfied n (%)	Somewhat satisfied n (%)	Somewhat dissatisfied n (%)	Very dissatisfied n (%)	Missing data n
How have you felt about the service during the last 2 years?	Hospice A	42 (53.2)	29 (36.7)	6(7.6)	2(2.5)	2
	Hospice B	64 (71.1)	21 (23.3)	0(0)	5(5.6)	1
How have you felt about the availability of practical support and guidance?	Hospice A	38(48.1)	30 (38.0)	10(12.7)	1(1.3)	2
	Hospice B	56(62.2)	29 (32.2)	1(1.1)	4(4.4)	1
How have you felt about communication with the hospice?	Hospice A	33(42.3)	26(33.3)	16(20.5)	3(3.8)	3
	Hospice B	49(55.7)	32(36.4)	3(3.4)	4(4.5)	3
How have you felt about prescribing practices?	Hospice A	36(47.4)	23(30.3)	11(14.5)	6(7.9)	5
	Hospice B	52(59.1)	29(33.0)	3(3.4)	4(4.5)	3
How have you felt about your level of involvement once the hospice is involved?	Hospice A	37(48.7)	32(42.1)	6(7.9)	1(1.3)	5
	Hospice B	51(59.3)	28(32.6)	4(4.7)	3(3.5)	5

An overall satisfaction index was created for both Hospice A and Hospice B.

**Figure 1:**
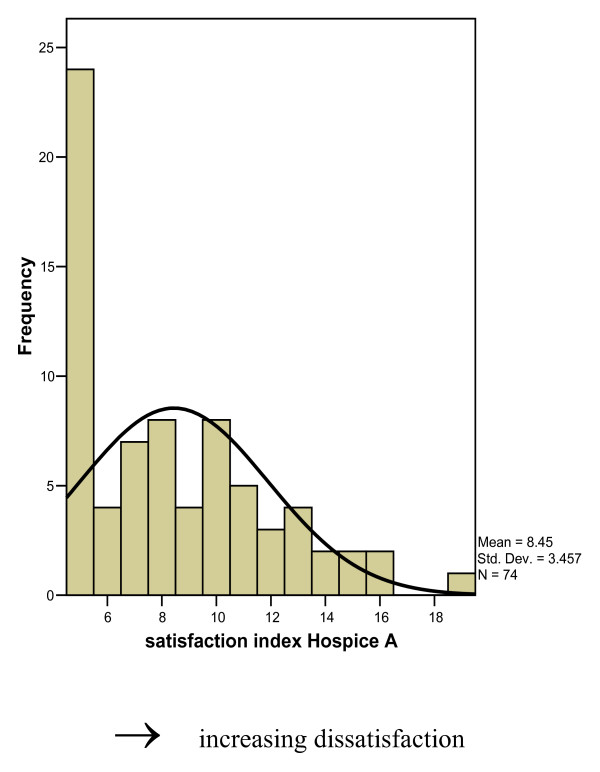
Overall index of satisfaction expressed by GPs for Hospice A.

**Figure 2:**
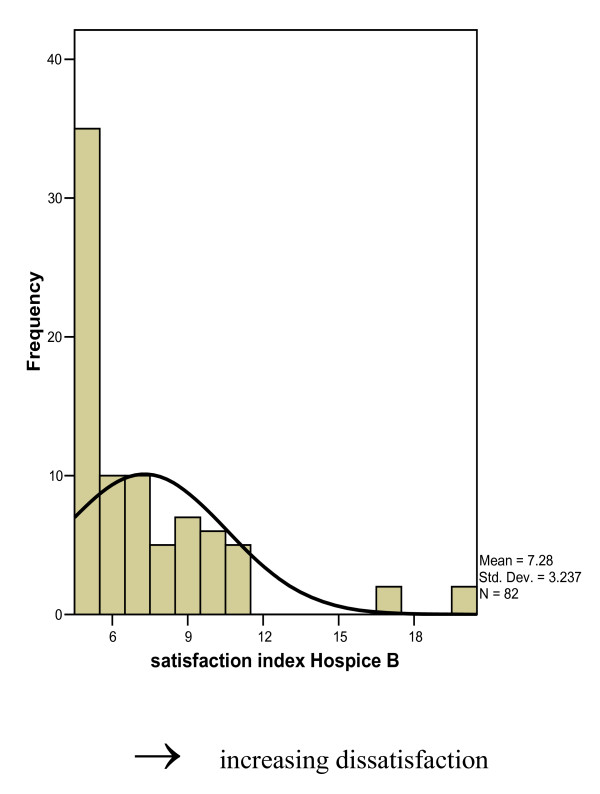
Overall index of satisfaction expressed by GPs for Hospice B.

The lower the satisfaction index, the more satisfied the GP was with the quality of the service. (Figures 1 and 2). Both histograms show skewed J shaped distributions with the most respondent being most satisfied (mode 5.0).

No significant relationship was found between the overall satisfaction levels for the specialist services and gender, age, number of partners, years as GP, practice size or the percentage of patients whose palliative care needs could be controlled without the help of the palliative care services.

There did however appear to be a relationship between trainer status and overall satisfaction levels. There was a non-significant tendency for trainers to be less satisfied with Hospice A than non-trainers (χ^2 ^= 2.93, df = 1, p = 0.09).

The most common number of patients referred by the GPs in the last 2 years was 1–3. (31 (40%) Hospice A users, Hospice B users (n = 37, 44%)). Greater than 80% of the users of the services had referred less than 7 patients to the hospices in the last two years- (83% and 82% for Hospice A and Hospice B respectively). There was no significant relationship between the overall satisfaction index of either hospices and the number of patients referred.

### Why are GPs not referring to the service?

**Figure 3:**
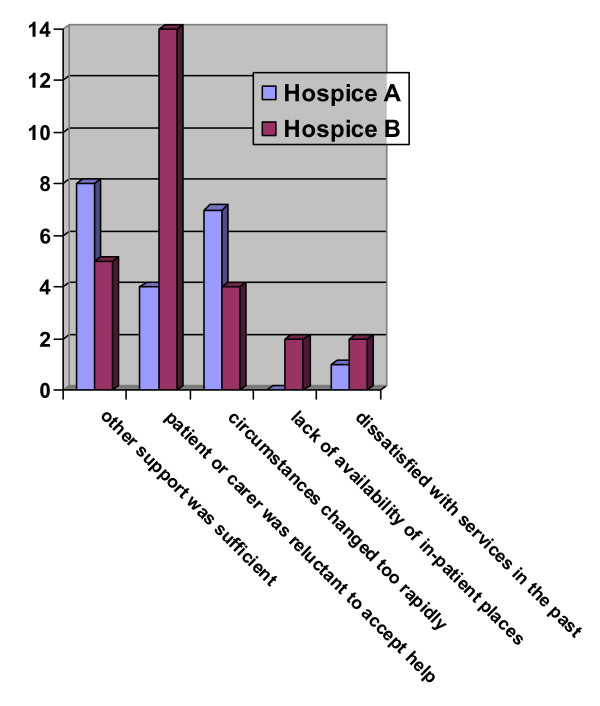
GPs' reasons for not referring to the specialist services.

13% (n = 11) of the Hospice A and 20% (n = 17) of Hospice B GPs (missing data n = 5) had had someone who was suitable for referral whom they had not referred. The most common reason for Hospice A users was that other support was sufficient (Figure 3). 14 (82%) of the Hospice B GPs not referring a patient, did not refer because the patient or carer was unwilling to accept help. Only 1 and 2 users of Hospice A and Hospice B respectively, did not refer a patient because they had been dissatisfied with the hospice services in the past.

A very high proportion of GPs-76 (96%) using Hospice A and 91 (98%) of the GPs using Hospice B would recommend the service to a colleague. Only 2 GPs did not complete this part of the questionnaire, leading to missing data.

### Comments section

**Figure 4:**
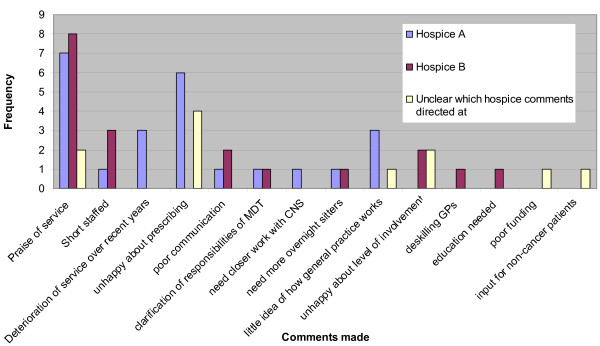
Comments made about services.

A comments section was included at the end of the questionnaire. 24 comments were made in total with regards to Hospice A. 7 comments praised the service and 17 criticised (Figure 4).

19 comments were made with regards to Hospice B. 42% of these comments were praising the service and included "excellent" and "very good".

The most common criticism was with regards to staffing issues at the hospice (n = 3, 27% of criticisms).

11 comments were made by GPs who had experience of both Hospice A and Hospice B with no clear indication at whom these comments were addressed.

2 of these 11 comments praised the services. The most common complaint was with regards to prescribing issues (44% of criticisms made towards the services). These included "the services prescribe too much" and " [we] need written instructions on drug changes".

## Discussion

The overall response rate and the percentage of questionnaires available for analysis here are better than the usual 61% in published studies involving GPs [12]. It is possible, that having the survey conducted by a GP registrar working in the area improved the response.

Seven GPs did not want to take part in the survey: two GPs stating that they did not partake in any surveys. Forty two GPs did not reply despite 3 questionnaires being sent out to them. An earlier study in north London [13], found the lowest level of awareness of available palliative care services was among non-responders. Non-responders in this study may also have a lower awareness of service.

The sex and age of the sample was similar to that of the GP principals in the PCT. Demographics provided by the Health Authority did not include those for salaried GPs. However, we felt that it was important to include salaried GPs in the sample to get full representation of views of the permanent GPs working in the area. This may have influenced the results obtained.

Single handed practices who all have list sizes less than 5000, were under-represented in the sample. It is difficult to assess from this data whether these GPs are using the specialist services differently than GPs supported by other colleagues. All trainers in the area responded to the questionnaire.

The majority of GPs felt that less than 1 in 4 cases could be managed without the help of the specialist services. It is unclear whether this reflects a high regard for the local services, a lack of confidence in symptom control management, or a wish to share care with other experts. Very few GPs felt that 100% of the patients could be managed without the help of the specialist services. Trainers were more likely to feel that patients could be managed satisfactorily without the help of the specialist services. Haines et al [14] found in their study that there was a slight but consistent tendency for general practitioners who had looked after four or more patients with a terminal illness in the past year to have fewer problems with the control of symptoms. It may be that trainer GPs are more experienced or trained in palliative care symptom management. However, this should be interpreted with caution. Haines et al [14] also found that there was no significant association between year of graduation and problems experienced. In this survey, there was no significant relationship between years as GP and percentage of cases whose palliative care needs could be satisfactorily controlled without the help of the specialist services. However, this study suggests that, according to GPs, on average 3:4 patients who GPs perceive as having progressive illness, are also thought to need specialist palliative care. In addition, of interest would be patients not recognised by GPs as requiring specialist palliative care input- in particular the non-cancer diagnoses. This survey highlights a possible need for specialist palliative care input which is higher than current levels of provision. This may be important for future needs assessment and would require a substantial increase in specialist services if it were to be provided.

GPs are most commonly using the services to work with them as part of an extended team. The study by Shipman et al [6] found that 14% of GPs seldom used the specialist service which is higher than the 8% in this survey. This may be related to the areas covered. Shipman et al [6] included inner city, urban and rural areas and fewer (only 8%) GPs worked with specialist services as part of an extended team, compared to greater than 65% in our study. We found a significant relationship between how GPs were using the hospice services and how they would like to use them; showing that most people were using the services in the way that they wished to.

Boyd et al [3] found that, 1 out of 5 GPs wanted specialist services to take over the care of the patient. Our study found a higher figure – 2 out of 5. However, Boyd et al [3] studied cases in Hackney, Tower Hamlets and Newham, a multi-racial, inner London area with multiple deprivation [15]. With a population of this kind, it is possible that GPs were less agreeable to referral. The new General Medical Services contract for primary care in England had not prioritised palliative care in its points allocation at the time of this research, therefore it is possible that other quality indicators were taking precedence. As a result, perhaps more GPs preferred to hand over care to the specialist services than had previously.

Boyd et al [3]also found that 75% of GPs wanted shared medical care; this is similar to our findings.

Our survey reflects a very high regard for the two palliative care services.

Lloyd et al [4] found that 70% of GPs felt that communication with the specialist services was very good or good and 80% of GPs in the Seamark et al's [5] survey felt that communication concerning the patients progress was sufficient. This is similar to that found for our specialist services where greater than 75% of the GP users were very/somewhat satisfied with the level of communication.

Greater than 75% of GPs using the service were satisfied with the prescribing practices of the specialist services with some variation between the two hospices.

In the US, Ogle et al [8], found that there was no significant feeling of loss of control over the management of the patient experienced by the primary care physician. Similar results were found in this study, with over 90% of users being very satisfied or satisfied with their level of involvement once the specialist services were involved.

GPs identified some patients who they had not referred. Most commonly where patients or carers were reluctant to accept help, or other support was deemed sufficient. Less than 2% of the GPs did not refer patients because they were dissatisfied with the service in the past. This is lower than the 30% in the Cambridge hospital at home study [11]. There was also no significant relationship between the overall satisfaction index for either hospice, or the number of patients referred to the service. This suggests that any dissatisfaction with the hospice services was not seen as a barrier to referral as in the US study by Ogle et al [8].

Seamark et al [5] found that 1 in 10 GPs replying to their questionnaire, felt that when the specialist service was involved, they found it difficult to know who had overall responsibility for patient care. In our study, this was higher. Clarification of the rules and responsibilities of the MDT was wanted by approximately 1 in 3 GPs. Whether this is related to increased accessibility of members of the multi-disciplinary team in a less rural environment is unclear.

Field et al [2] showed that GPs wanted to call in expert help as required but not to surrender care to the palliative care services, and our findings support this. Although overall satisfaction with the service was high, greater than 50% of GPs using the services felt there were areas where improvement could be made, with clarification of the rules and responsibilities of the MDT being the most common.

A number of comments related to a lack of perception by the specialist services as to how general practice works, were unexpected and not noted in the literature reviewed. Other areas of possible improvement included communication and prescribing practices. However, both hospices received praise in the comments section showing a great appreciation of the services available.

Greater than 90% of the users of both specialist services would recommend the service to a colleague despite any dissatisfaction expressed. Overall GPs are very satisfied with the services.

There were a number of limitations to this study. The nature of the design of the study allowed assessment of views at one moment in time, but some GPs replied late or only in response to reminders. The data obtained from such a short questionnaire is limited, but data richness was compromised as we believed that a short questionnaire would have a higher response rate. In addition to this, our study was in one part of England, and although it shows similarity with some other published work and was in an area of slightly better than average affluence, may not be fully representative of other populations, especially of inner city areas.

## Conclusion

As primary care physicians have taken on a more significant role in hospice referral and ongoing care, their role as potential impediment to palliative care services has been questioned [8].

Many GPs did not feel that any improvement was needed in the service provision of these two hospices in the south of England. However, the majority of GPs felt that 3 out of 4 patients in the community required specialist palliative care input. This may be important in planning future service provision.

More GPs would like to hand over care of these patients to the specialist service. The significance of these findings to recent changes in general practice in the UK remain unclear and requires further investigation.

This study, having achieved a good response rate, found that between 13 and 20% of GPs had had patients suitable for referral that they had not referred. However, for the majority of GPs, this was due to reasons unrelated to the hospice services. A further area of study would include investigating the understanding of the specialist services into how general practice works.

## Competing interests

During the time the research was conducted, SB was independent of both hospices.

Subsequently, after the research was completed, SB underwent a placement at hospice B. Neither of the hospices had any input into the study but have been subsequently presented with the results.

## Authors' contributions

SB conceived of the study, designed and coordinated, performed the statistical analysis and drafted the manuscript. IH has have been involved in revising the manuscript critically for important intellectual content and has given approval of the version to be published.

## Pre-publication history

The pre-publication history for this paper can be accessed here:



## References

[B1] Field D, James N, Clark D (1993). Where and how people die. The future for palliative care; issues in policy and practice.

[B2] Field D (1998). Special not different: General practitioners' accounts of their care of dying people. Soc Sci Med.

[B3] Boyd K (1993). Palliative care in the community: Views of General Practitioners and District Nurses in East London. J Palliat Care.

[B4] Lloyd-Williams M, Wilkinson C, Lloyd-Williams F (2000). General practitioners in North Wales: current experiences of palliative care. Eur J Cancer Care.

[B5] Seamark DA, Thorne CP, Jones RVH, Pereira Gray DJ, Searle JF (1993). Knowledge and perceptions of a domiciliary hospice service among general practioners and community nurses. Br J Gen Pract.

[B6] Shipman C, Addington-Hall J, Barclay S, Briggs J, Cox I, Daniels L, Millar D (2002). How and why do GPs use specialist palliative care services?. Palliat Med.

[B7] Desmedt M, Michel H (2002). Palliative home care: improving co-operation between the specialist team and the family doctor. Support Care Cancer.

[B8] Ogle K, Mavis B, Wang T (2003). Hospice and primary care physicians: attitudes, knowledge and barriers. Am J Hospice and Palliat Care.

[B9] Groot M, Vernooij-Dassen M, Verhagen S, Crul B, Grol R (2007). Obstacles to the delivery of primary palliative care as perceived by GPs. Palliat Med.

[B10] Cartwright A (1991). Balance of care for the dying between hospitals and the community: perceptions of general practioners, hospital consultants, community nurses and relatives. Br J Gen Pract.

[B11] Todd CJ, Grande GE, Barclay SI, Farquhar MC (2002). General practioners' and district nurses' views of hospital at home for palliative care. Palliat Med.

[B12] Sibbald B, Addington-Hall J, Brenneman D, Freeling P (1994). Telephone versus postal surveys of general practioners: methodological considerations. Br J Gen Pract.

[B13] Higginson I (1999). Palliative care services in the community: what do family doctors want?. J Palliat Care.

[B14] Haines A, Booroff A (1986). Terminal care at home: perspective from general practice. Br Med J.

[B15] Office of Population Censuses and Surveys Mortality statistics for 1978 and 1987. England and Wales.

